# Bridging Glycemic and Lipid Control: Effects of Dapagliflozin in Co-Morbid Diabetes and Heart Failure – A Randomized Controlled Trial

**DOI:** 10.2174/0118715303398749251027044323

**Published:** 2026-01-15

**Authors:** Laxmi Sankalpa Bhaganagarapu, Sarumathy Sundararajan, Vengatesh Munusamy

**Affiliations:** 1 Department of Pharmacy Practice, SRM College of Pharmacy, Faculty of Medicine and Health Sciences, SRM Institute of Science and Technology, Kattankulathur, Chengalpattu dist. Tamil Nadu , 603203, India ;; 2 Department of Cardiology, SRM Medical College Hospital and Research Centre, Faculty of Medicine and Health Sciences, SRM Institute of Science and Technology, Kattankulathur, Chengalpattu dist. Tamil Nadu, 603203, India

**Keywords:** Dyslipidemia, co-morbid diabetes, dapagliflozin, heart failure, insulin resistance, SGLT2 inhibitor

## Abstract

**Introduction:**

Dapagliflozin, an SGLT2 inhibitor developed for Type 2 Diabetes Mellitus (T2DM), has shown multiple cardiovascular benefits. This study evaluated its effects on lipid profile and IR pattern in South Indian patients with T_2_DM and Heart Failure (HF).

**Methods:**

This randomized trial was conducted over 180 days in 58 patients with T2DM and HF, assigned in a 1:1 ratio to standard therapy or standard therapy plus dapagliflozin 10 mg daily. Lipid, glycemic, and IR parameters were assessed at baseline, 12 weeks, and 24 weeks.

**Results:**

Dapagliflozin group showed marked improvements in BMI (*p* = 0.01), TC (*p* = 0.02), TG (*p* = 0.01), HDL (*p* = 0.001), LDL (*p* = 0.002), and VLDL (*p* = 0.01), FPG (*p* = 0.0001), PPG (*p* = 0.002) and HbA1c (*p* = 0.01), HOMA-IR (*p* = 0.002), HOMA2%B (*p* = 0.003), and decreased insulin levels (*p* = 0.001) by 24 weeks. In contrast, significant changes were noted only in LDL (*p* = 0.01), HDL (*p* = 0.03), and TG (*p* = 0.01) in the standard group. A positive correlation was observed between the mean change in TG and HOMA-IR after 24 weeks of dapagliflozin treatment.

**Discussion:**

Dapagliflozin improved lipid profile and reduced IR, demonstrating benefits in T2DM and HF patients, which aligned with the findings from the larger SGLT2i trials. Study limitations included the small sample size, single-center setting, and an open-label design.

**Conclusion:**

Dapagliflozin improved lipid and IR, highlighting its potential as an adjunctive therapy in patients with co-morbid T2DM and HF.

**Clinical Trial Number:**

CTRI/2024/01/06208.

## 1 INTRODUCTION

Globally, the prevalence of Type II Diabetes Mellitus (T2DM) has risen exponentially from 537 million adults worldwide in 2021 to 589 million in 2024, and is projected to reach 853 million by 2050, indicating a substantial health care burden as per the International Diabetes Federation [1, 2]. These statistics emphasize the need for effective prevention and treatment strategies to manage T2DM and its co- morbidities. Cardiometabolic diseases continue to be the leading cause of mortality worldwide, with approximately 60% of the disease burden affecting individuals in the Asia-Pacific region [3]. The prevalence of T2DM among adults in India increased 2.2-fold from 33 million in 2000 to 72 million in 2021, with an expected rise to 125 million by 2045, projecting a 3.8-fold spike [4]. India has emerged as an epicenter of global T2DM, with a rapid rise among young individuals, underpinned by reduced beta-cell function, low lean body mass, and visceral fat accumulation, and additionally influenced by genetics and sedentary lifestyle [5, 6].

Dyslipidemia is one of the prevalent comorbidities in T2DM, characterized by abnormal lipid profiles, particularly elevated levels of small dense particles of Low-Density Lipoprotein (sd-LDL), Triglycerides (TG), and decreased levels of High-Density Lipoprotein (HDL), raising the risk of cardiovascular death [7, 8] The advancement of Heart Failure (HF) is mainly influenced by hyperglycemia and abnormal lipid profiles [9, 10]. The interaction of hyperglycemia and dyslipidemia in the development of HF is intricate and multifactorial, arising from Insulin Resistance (IR), oxidative stress, chronic inflammation, endothelial dysfunction, and lipotoxicity [9, 11]. Reduced insulin secretion and sensitivity in T2DM are closely linked to dysregulation of lipid profiles [12, 13]. Several pathways link IR with lipid metabolism, particularly contributing to hypertriglyceridemia [14]. It is predominantly mediated by excess production of Very Low-Density Lipoprotein (VLDL) in the liver and raised apo lipoprotein B-100 (apo-B100) production, which acts as an integral component of VLDL [15]. IR can also activate Hormone Sensitive Lipase (HSL), an enzyme that increases the release of Free Fatty Acids (FFAs) into circulation, by the hydrolysis of TGs [16]. Excess FFAs enter non-adipose tissues and accumulate as ceramide or DAG, leading to lipotoxicity. This storage can disrupt the normal insulin signalling [16, 17]. Elevated HSL activity can also enhance hepatic VLDL production, thereby intensifying dyslipidemia [16].

Some studies have suggested that under normal glycemic conditions, insulin inhibits VLDL release, but in IR, the liver becomes less responsive, leading to elevated VLDL levels regardless of insulin levels [18]. Moreover, IR reduces the activity of Lipoprotein Lipase (LPL), a key enzyme that breaks down VLDL in skeletal muscle and adipose tissue. In conditions of IR, LPL activity diminishes, impairing VLDL clearance. Subsequently, IR enhances lipolysis and reduces fat absorption by adipocytes, resulting in higher circulating FFA levels [19]. These excess FFAs are delivered to the liver and intestine, leading to the overproduction of TG-rich lipoproteins [18].

SGLT2 inhibitors (SGLT2i), a newer class of medication for T2DM management, act by selectively blocking the SGLT2 protein responsible for glucose reabsorption in the renal tubules, thereby helping lower blood glucose levels. Beyond glycemic control, these agents provide a range of beneficial metabolic and vascular effects, including enhanced hemoglobin levels, reduced dyslipidemia, lowered blood pressure, and enhanced antioxidant properties [20, 21]. Although the DAPA-HF (Dapagliflozin and Prevention of Adverse Outcomes in Heart Failure) and EMPEROR-Reduced trial (Empagliflozin Outcome trial in patients with Chronic Heart failure with reduced ejection fraction), conclusively demonstrated that SGLT2i significantly reduces the risk of hospitalizations and cardiovascular death in patients with HF, they did not focus on the lipid parameters as part of their outcomes [22, 23]. However, only a few studies have focused on the dyslipidemic effects of SGLT2i beyond glycemic control [24, 25]. Some hypotheses suggest that the lipid-lowering effect might be due to enhanced fatty acid oxidation through pathways involving AMP-Activated Protein Kinase (AMPK) and Peroxisome Proliferator-Activated Receptor-alpha (PPAR-α) activation, which also modulate LPL activity [26]. However, the mechanisms underlying these effects are not yet fully understood.

Despite the glycemic effects and cardioprotectant effects of dapagliflozin being well established, its interlinked metabolic effects, particularly on dyslipidemia and IR, remain less explored, particularly in the Indian population. Therefore, this study was designed to address this gap by assessing the effects of dapagliflozin on IR and lipid parameters in South Indian patients with co-existing T2DM and HF.

## 2 METHODOLOGY

### 2.1 Study Design

This open-label, randomized controlled prospective study was conducted on 58 patients for six months (June 2024 to December 2024) in the Cardiology unit of a tertiary care hospital. The trial received ethical clearance from the Institutional Human Ethics Committee of SRM Medical College Hospital and Research Centre, Kattankulathur, Tamil Nadu, India (*Ref.no: 8522/IEC/2023)*. The study followed the guidelines proposed in the Declaration of Helsinki and was registered with the Clinical Trial Registry of India (CTRI) *(Ref no: CTRI/2024/01/06208).*

### 2.2 Inclusion Criteria and Exclusion Criteria

The study included clinically stable patients with T2DM and HF of either sex aged between 20-80 years with HbA1c (> 6.7%), reduced ejection fraction (EF ≤ 40%) or preserved ejection fraction (EF ≥ 50%), abnormally elevated lipid profile parameters such as Total Cholesterol (TC) (>200 mg/dl), TG (>150 mg/dl), VLDL (>30 mg/dl), LDL (>130 mg/dl) and decreased HDL (<40 mg/dl). Patients with HOMA-IR ranges above 2.73 were considered to have IR and were included [27]. HF patients with T2DM who were on a standard treatment regimen and had voluntarily provided written informed consent to participate were eligible for inclusion in the study.

Patients were excluded if they had received treatment with any SGLT2 inhibitor within one month prior to enrollment, were on high doses of lipid-lowering therapy (such as statins or fibrates) for uncontrolled dyslipidemia, had uncontrolled T2DM (HbA1c > 10%) or a history of diabetic ketoacidosis, had severe comorbid conditions including end-stage renal disease (GFR < 15 mL/min/1.73 m^2^), or had severe hepatic impairment. Individuals with untreated endocrine disorders such as hypothyroidism or Cushing’s syndrome, lipid-related malabsorption syndromes, patients with a history of recurrent urinary tract infections, as well as pregnant or breastfeeding women, were also excluded.

### 2.3 Sample Size Determination

The sample size was estimated using the standard formula for a randomized controlled trial, with a 95% Confidence Interval (CI) and a power of 80% (1-β). Based on the standard deviation (SD) reported in previous research articles relevant to the study, as 8.2 [28], the sample size was calculated to be 29 participants per treatment group. A total of 5 patients were lost to follow-up, resulting in an attrition rate of 8.5%. The study's adjusted power was 73%.

### 2.4 Randomization

A total of 75 patients with T2DM and HF were selected from the Cardiology unit, of whom 58 patients met the study’s requirement criteria. They were randomly allocated to two groups, namely i) the Standard treatment group (n = 29) and ii) the Intervention group (n = 29). During the follow-up period, two patients in the standard arm were relocated, and three in the intervention arm (two patients were lost to contact, and one patient relocated) were unavailable to continue the study. Hence, the total analyzed cohort comprised 53 patients, with 27 in the standard group and 26 in the treatment group. The randomization was performed using an open list of random numbers generated by a random allocation software tool. A third person unrelated to the study generated the allocation sequence.

### 2.5 Procedure of the Study

Before enrollment, a clear explanation of the study procedure was provided, and each patient then signed an informed consent document. Patients who fulfilled the inclusion criteria were then randomized into two groups. The ‘standard treatment group’ received the conventional HF treatment, including beta-adrenergic blockers, diuretics, Angiotensin Converting Enzyme inhibitors (ACEi), Angiotensin Receptor Blockers (ARBs), HMG Co-A reductase inhibitors, positive inotropic agents, and oral hypoglycemic agents (biguanides or sulfonylureas) as prescribed by the physician. The ‘intervention group’ received the standard treatment along with dapagliflozin 10 mg/orally/once a day for 24 weeks. Blood glucose parameters such as PPG (Postprandial Plasma Glucose), HbA1c, and FPG (Fasting Plasma Glucose), IR indices namely fasting insulin, Homeostatic Model Assessment of Insulin Resistance (HOMA-IR), and Homeostatic Model Assessment of β-cell function (HOMA 2%B) and lipid profile parameters like LDL, TC, HDL, TG, and VLDL were assessed at the baseline, end of 12^th^ week and 24^th^ week of treatment. Dapagliflozin was dispensed to patients at each visit, and both study groups were counselled on the importance of medication adherence. The consort flowchart for the research study is shown in Fig. (**1**).

### 2.6 Anthropometric Parameters

The anthropometric parameters, such as Blood Pressure (BP), height, weight, and BMI, were assessed at each visit in the study population. The patient’s BP was measured by a digital sphygmomanometer (Diamond, BPDG234, Mercury-free LCD Super deluxe BP monitor). Two BP readings were taken at 10-minute intervals, and the average of the two consecutive measurements was recorded as the mean BP. The patient’s height was measured barefoot and recorded. Weight was measured using a weighing machine (Equinox’s mechanical weighing scale Eq-Br-9201). BMI was calculated using the formula weight/height^2^ (kg/m^2^). The patients were categorized into three different BMI categories such as normal, overweight, and obese [29]. Insulin resistance and beta cell function were calculated using HOMA-2 online calculator (https://www.dtu.ox.ac.uk/homacalculator).

### 2.7 Biochemical Analysis

After 10-12 hours of overnight fasting, 6 ml of venous blood was collected from each patient.

From this, 2 ml was centrifuged for 10 minutes at 3000-4000 rpm to separate plasma, and FPG estimation was done using the enzymatic hexokinase method. Another 2 ml blood sample was centrifuged to estimate fasting insulin by immunoassay and lipid profile by enzymatic analysis. A further 2 ml of the remaining blood sample was processed for HbA1c testing using the HPLC method. Additionally, 2 ml of the blood sample was collected 2 hours post-meal and centrifuged to separate plasma for PPG analysis.

### 2.8 Statistical Interpretation

GraphPad Prism software (V8, San Diego, CA) and SPSS (V24, SPSS Inc., Chicago, IL, USA) were employed for statistical analysis. The data were presented as the mean ± standard deviation changes. The mean changes in the parameters within the treatment groups at different time points were compared using a repeated-measures ANOVA with multiple comparisons. The baseline changes were analyzed using an unpaired t-test. ANOVA assessed between-group changes at 12 and 24 weeks. Statistical significance is indicated by a *p*-value less than 0.05.

### 2.9 Safety Profile

The study participants were encouraged to report any untoward symptoms, discomfort, or any health issues encountered throughout the study. At each follow-up visit, they were asked to report any such events. All the observed abnormalities were thoroughly examined and documented by the study investigators.

## 3 RESULTS

### 3.1 Pre-treatment Values

The mean age in the standard group was 61 ± 5.9 years with an average BMI of 29.0 ± 1.4 kg/m^2,^ and the intervention group was 62.1 ± 7.6 years with a BMI of 31.0 ± 3.2 kg/m^2^. Most of the patients were either obese or overweight based on their BMI. There were no significant differences between the groups in baseline characteristics (Table **1**).

### 3.2 Post-treatment Changes in Glycemic Parameters Within the Groups

In the standard group, a statistically significant reduction in FPG (*p* = 0.001) and PPG (*p* = 0.01) was observed after 24 weeks compared to baseline.

In the intervention group, significant reduction in FPG (*p* = 0.0001), PPG (*p* = 0.002), HbA1c (*p* = 0.01), HOMA-IR (*p* = 0.002), fasting insulin (*p* = 0.001) and an increase in HOMA-2%B (*p* = 0.003), were noted compared to the baseline values after 24 weeks of treatment (Table **2**).

The represented values were expressed as mean ± standard deviation changes. *P*-values were calculated based on repeated measures ANOVA for multiple comparisons.

### 3.3 Post-treatment Changes in Lipid Parameters Within the Groups

In the standard group, after 24 weeks of treatment, a statistically significant improvement in HDL (*p* = 0.03) and a decrease in LDL (*p* = 0.01) and TG (*p* = 0.01) were noted compared to their baseline values. In the intervention group, significant reductions were noted in TC (*p* = 0.02), LDL (*p* = 0.002), VLDL (*p* = 0.01), TG (*p* = 0.01), and BMI (*p* = 0.01). A significant increase in HDL levels (*p* = 0.001) was also observed after 24 weeks of treatment compared to their baseline values (Table **3**).

### 3.4 Between-group Comparisons

There was a significant improvement in glycemic control in the intervention group after 12 weeks, as indicated by FPG and PPG (*p* = 0.001) and HbA1c (*p* = 0.01). Significant reductions were noted in FPG and PPG (*p* = 0.001) and HbA1c (*p* = 0.009), fasting insulin levels (*p* = 0.02), and an improvement in HOMA2%B (*p* = 0.01) after 24 weeks of dapagliflozin treatment compared with standard treatment alone.

Lipid parameters were significantly improved in the intervention group. HDL showed a significant increase (*p* = 0.02), while TG levels decreased (*p* = 0.03). By 24 weeks, there was a significant improvement in HDL (*p* = 0.01), and a marked reduction in TC and LDL (*p* = 0.001), VLDL (*p* = 0.03), TG (*p* = 0.01), and BMI (*p* = 0.02) was observed after 24 weeks of dapagliflozin treatment in comparison to standard treatment alone (Table **4**).

### 3.5 Pattern Of Correlation Between TG *Vs* HOMA-IR

**Fig (2):**
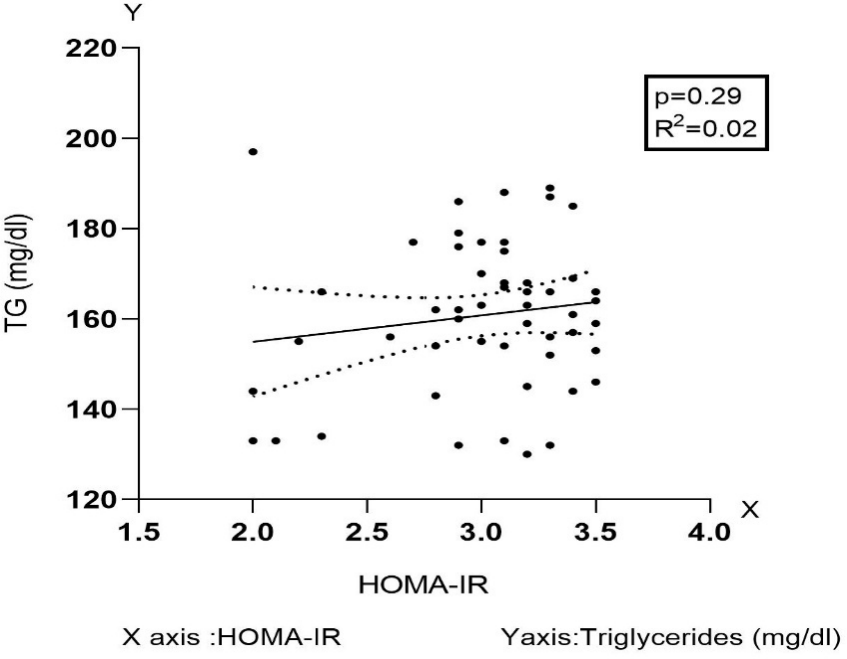
Correlation between TG *Vs* HOMA-IR at baseline in both the treatment groups.

Pearson’s correlation analysis was performed to assess the association between the mean change in TG and the mean change in HOMA-IR at 24 weeks in both groups. At the baseline visit, there was no significant correlation between TG *Vs* HOMA-IR in the overall study population (Pearson correlation coefficient (*r*) = 0.14; Coefficient of determination (*R^2^*) = 0.02, *p* = 0.29; 95% Confidence interval (CI): –0.12 to 0.4) (Fig. **2**).

**Fig (3):**
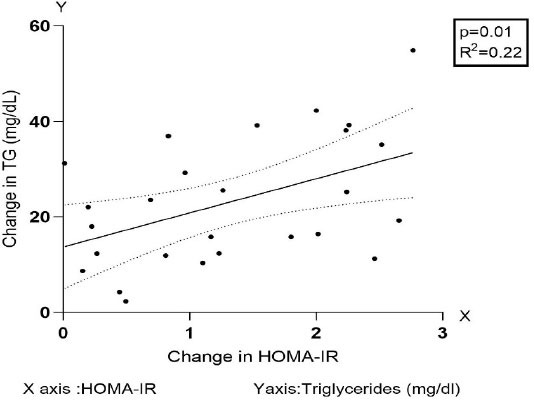
Correlation between mean change in TG *Vs* mean change in HOMA-IR after 24 weeks in the intervention group.

Following 24 weeks of treatment, the intervention group demonstrated a statistically significant positive correlation between mean change in TG *Vs* HOMA-IR after 24 weeks of treatment (*r* = 0.47, *R^2^* = 0.22, *p* = 0.01; 95% CI: 0.18 to 0.72), indicating a strong association between these parameters (Fig. **3**).

**Fig (4):**
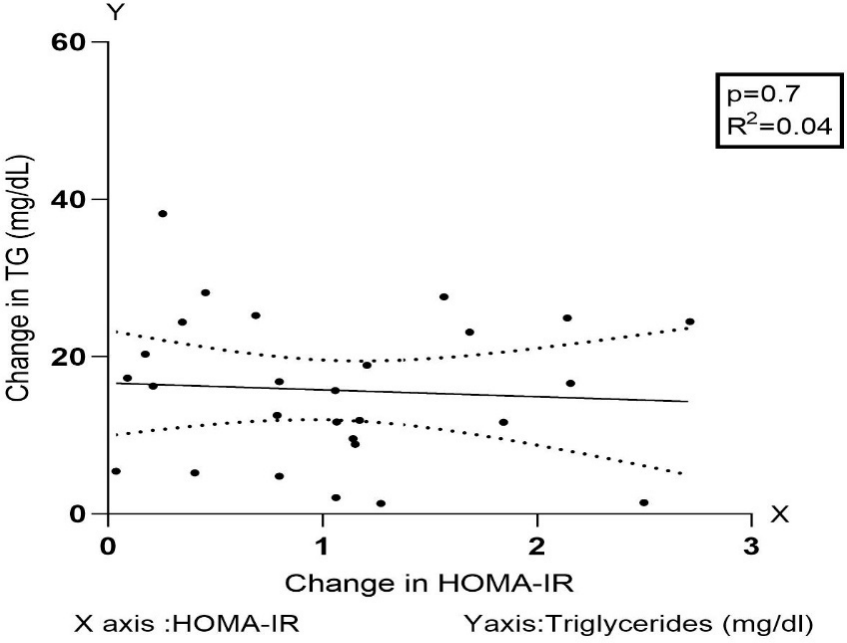
Correlation between mean change in TG *Vs* mean change in HOMA-IR after 24 weeks in the standard group.

In contrast, the standard group showed a very weak and non-significant correlation (*r* = –0.06, *R^2^* = 0.04, *p* = 0.7; 95% CI: -0.43 to 0.31), reflecting no significant correlation between these parameters after 24 weeks of standard therapy (Fig. **4**).

### 3.6 Side Effects Observed in Both Treatment Arms

Few side effects, such as mild UTI presented as burning micturition (n=5) and electrolyte imbalances (n=2), were noted in the intervention group. Gastrointestinal disturbances (n=5), hypokalemia (n=3), and palpitations (n=2) were noted in the standard group.

T2DM patients are typically more prone to develop infections, particularly UTIs, due to inadequate glycemic control [30]. The mechanism of glucosuria-mediated glycemic control by SGLT2i creates a favorable environment in the urinary tract for microbial growth, leading to an increased risk of UTIs. These side effects are usually managed with antibiotic therapy and do not require discontinuation of dapagliflozin [31]. The patients received necessary medical care to manage these side effects. Electrolyte imbalances were corrected with oral supplements.

## 4 DISCUSSION

The current research was designed to evaluate the dyslipidemic, glycemic implications, and IR pattern associated with the integration of dapagliflozin into the conventional treatment regimen in patients with co-morbid T2DM and HF. In T2DM, the process of inflammation and oxidative stress can be accelerated by dyslipidemia and an increased IR, which can potentially contribute to the early onset of HF [32]. Data from large clinical trials in T2DM have shown that SGLT2i therapy reduces the risk of hospitalization and prevents early-onset [22, 23]. These can be attributed to mechanisms beyond glucose reduction, such as decreased volume overload, decreased lipotoxicity, preserved kidney function, minimised myocardial fibrosis, and many more, which can collectively contribute to the cardio-protectant effect in HF patients [20]. Dapagliflozin has a C-glucoside structure, in which a glucose is attached to an aryl group *via* a carbon-carbon bond, thereby ensuring selectivity for SGLT-2 [33]. The dyslipidemic effect observed with SGLT2i might result from secondary metabolic effects, such as weight loss and improved insulin sensitivity [20]. Dapagliflozin provides a supportive role in lipid management, but its combination with conventional lipid-lowering drugs is essential to achieve recommended lipid targets in dyslipidemia.

The average age of the patients in this study was 61 years, with the majority of them being obese with a mean BMI of 30 kg/m^2^. The prevalence of overweight and obesity has risen in recent times, exerting a significant negative impact on health outcomes [34]. According to Shen *et al*. [35], obesity is an early prognostic indicator of HF. Hence, the primary treatment strategies for HF should address the underlying obesity, followed by the management of cardiovascular complications. As per our research findings, the dapagliflozin group had a significant reduction in BMI than the conventional treatment alone after 24 weeks of treatment. The probable reduction in body mass might be due to glucosuria, which creates a calorie-deficient state, promoting weight loss, although the exact mechanism remains unclear [36, 37].

The glycemic parameters (FPG, PPG, and HbA1c) significantly improved, which was due to distinct glucosuric effects exerted by this group of drugs, as 90% of glucose reabsorption by the kidney is blocked by SGLT-2 inhibition at the proximal convoluted tubule, which can therefore alter glycemic indices [38-41]. Consistent with these findings, the between-group analysis showed significant improvements in FPG and PPG (*p*=0.001) and HbA1c (*p*=0.009) at 24 weeks of dapagliflozin therapy.

T2DM patients often present with obesity, in which adipose tissue primarily contributes to the development of IR by triggering inflammatory pathways, enhancing FFA releaseand disrupting normal adipokine function [13]. Pre-clinical evidence by Asahara *et al*. [42] concluded that SGLT2i preserved pancreatic islet structure, which was not observed with other antidiabetic agents like GLP-1 analogs or sulfonylureas, thereby improving pancreatic beta cell sensitivity. Gao *et al*. [43] also reported that SIRT1 (Sirtuin 1) activation under nutrient-deficient conditions played a critical role in skeletal muscle, leading to improved insulin sensitivity. Clinically, Veelen *et al*. [44] demonstrated that enhanced skeletal muscle mitochondrial oxidative capacity, along with increased fat oxidation, led to improved IR in pre-diabetic cases. The present study's findings are in line with previous studies showing that dapagliflozin improved IR and decreased fasting insulin levels, indicating reduced demand on pancreatic β-cells that could potentially preserve cellular function and reduce metabolic load. In the current study, there was a pattern of elevated IR, thereby affecting beta cell function, a common feature of T2DM. IR was assessed from HOMA-IR and HOMA-2%B values, which were substantially elevated at the baseline visit. After 180 days of SGLT2i treatment, improvements in fasting insulin (*p*=0.001), IR (*p*=0.002), and beta-cell function (*p*=0.003) were observed, highlighting the beneficial effects of dapagliflozin on insulin sensitivity.

In T2DM, TG levels are typically elevated due to the combined effects of IR and increased concentration of FFAs in the bloodstream. This leads to enhanced hepatic production of VLDL. Additionally, reduced LPL activity in peripheral tissues impairs the clearance of TG-rich lipoproteins, further contributing to hypertriglyceridemia [19]. The elevated TG levels promote the structural changes in LDL, resulting in a shift from large buoyant LDL (lb LDL) to small dense LDL (sd LDL) [45]. Higher levels of lipid parameters (LDL, TC, TG, VLDL) and a decrease in HDL levels are frequently encountered in T2DM, increasing cardiovascular risk [46, 47]. According to Hayashi *et al*. [25], in T2DM, dapagliflozin has been shown to lower sd-LDL, an atherogenic lipoprotein, while increasing lb-LDL-C, which is less likely to contribute to atherosclerosis. This accounted for an increase in total LDL-C, primarily due to increased lb-LDL. Additionally, dapagliflozin raises levels of HDL2, the cardio-protective form of HDL, without altering HDL3 form. In contrast, a study by Matthaei *et al*. [40] in T2DM patients concluded that dapagliflozin therapy over 24 weeks was associated with increases in TC and LDL-C, despite a reduction in TG levels. This discrepancy may be attributed to a rise in lb-LDL rather than sd-LDL, as well as differences in ethnicity, baseline lipid profiles, underlying treatment regimen, and duration. Moreover, the inclusion of statins in the present treatment regimen may have reinforced the LDL-lowering effect observed in our study.

The present study noted a significant change in the lipid profile after 24 weeks of dapagliflozin therapy. The between-group analysis at 24 weeks demonstrated significant improvements in lipid parameters, TC decreased by 11.6 mg/dL (*p*=0.001), LDL by 6.9 mg/dL (*p*=0.001), VLDL by 3.6 mg/dL (*p*=0.03), and TG by 9.6 mg/dL (*p*=0.01), and BMI by 4.0 kg/m^2^ (*p*=0.02). HDL increased by 4 mg/dL (*p*=0.02) when compared to the standard group. Our study aligned with the findings of the study by Calapkulu *et al*. [24], which concluded that dapagliflozin significantly improved glycemic parameters and decreased TC, LDL, and TG over 6 months, highlighting the need for long-term management.

A proportional rise in glucose concentrations and TG is frequently encountered in T2DM [48]. To explore the relationship between IR and TG, a correlation analysis was performed. A non-significant and weak positive correlation existed between IR and TG after 24 weeks of standard treatment. In contrast, a positive and significant correlation was observed after 24 weeks of dapagliflozin therapy, where a decrease in IR was associated with decreased TG levels. These findings suggest that dapagliflozin not only addresses glycemic control but also positively impacts lipid metabolism and IR, making it an effective therapeutic choice for patients with co-morbid T2DM patients with HF.

## CONCLUSION

This clinical study provides evidence that dapagliflozin, when added to the standard treatment regimen, was associated with significant improvements in lipid, glycemic, and IR parameters. A significant positive correlation was observed between mean HOMA-IR and mean TG values following 24 weeks of dapagliflozin therapy, suggesting a possible link between improvement in insulin sensitivity with TG levels. These findings suggest that dapagliflozin may play a beneficial role in managing dyslipidemia in patients with co-morbid T2DM and HF. However, larger studies are needed to clarify these results and evaluate their impact on long-term cardiovascular outcomes.

## LIMITATIONS OF THE STUDY

The small sample size limits the statistical power and may impact the subgroup analyzes and their associated clinical outcomes. As this was a single-center study conducted in a South Indian population, these findings cannot be generalized to a diverse population due to regional and genetic variations. The average age of the study population was 62 years, and further studies in other age groups are needed. Moreover, patients with an ejection fraction between 40% and 50% were excluded, limiting the applicability of the results to individuals with heart failure with mildly reduced ejection fraction (HFmrEF), a distinct subgroup of HF. It was challenging to verify mechanistic explanations for the observed changes in lipid profiles because metabolic pathways such as AMPK or PPAR-α activation were not evaluated in this study. These limitations highlight the need for future large-scale, multicentre studies involving more heterogeneous populations to validate the current findings. The study also observed higher standard deviations in glycemic and lipid parameters, which could be attributed to differences in the duration and severity of T2DM and HF, variations in medication use, lifestyle factors, and other individual-related differences that may have influenced the study outcomes.

## Figures and Tables

**Fig. (1):**
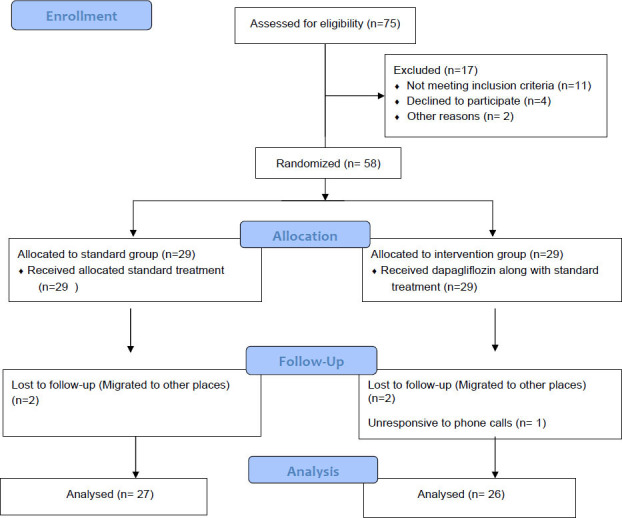
Consort flow chart of the study.

**Table 1:** Baseline characteristics of the study groups.

**Parameters**	**Standard Group (n=27)**	**Intervention Group (n=26)**	***P*-value**
Age (years)	61 ± 5.9	62.1 ± 7.6	0.42
Height (cm)	151.1 ± 4.30	155.3 ± 4.4	0.57
Body weight (kg)	75.1 ± 7.9	79.14 ± 6.9	0.08
BMI (kg/m^2^)	29.0 ± 1.4	31.0 ± 3.2	0.43
**Blood Pressure**			
Systolic BP (mm Hg)	132 ± 11.9	130.4 ± 16.9	0.76
Diastolic BP (mm Hg)	83.5 ± 6.5	83.8 ± 7.1	0.83
**Blood Glucose Parameters**			
FPG (mg/dl)	230.8 ± 41.2	253.2 ± 50.2	0.07
PPG (mg/dl)	304 ± 51.2	312 ± 67.2	0.26
HbA1c (%)	8.7 ± 1.3	8.5 ± 2.0	0.08
HOMA-IR	3.6 ± 1.27	3.8 ± 1.06	0.07
HOMA 2% B	68.16 ± 12.3	66.7 ± 14.7	0.09
Fasting Insulin (µIU/ml)	40 ± 3.45	41.2 ± 3.9	0.4
**Lipid Profile**			
TC (mg/dl)	153.9 ± 18.8	156.2 ± 14.9	0.61
LDL (mg/dl)	144.6 ± 11.8	148.7 ± 14.11	0.23
VLDL (mg/dl)	34.3 ± 3.55	35.5 ± 6.12	0.36
HDL (mg/dl)	37.5 ± 3.25	39.5 ± 6.07	0.12
TG (mg/dl)	168.1 ± 13.8	164.9 ± 13.4	0.37

**Note:** The represented values were expressed as mean ± standard deviation changes. *P*-values were calculated based on an unpaired t-test.

**Table 2:** Changes in the glycemic parameters within the treatment groups.

**Standard Treatment Group**						
**Glycemic Parameter**	**Baseline**	**12 Weeks**	**Mean Difference**	**24 Weeks**	**Mean Difference**	***P*-value**
FPG (mg/dl)	230.8 ± 41.2	197.8 ± 25.7	33 ± 36	164 ± 19.2	66.8 ± 35.7	0.001***
PPG (mg/dl)	304 ± 51.2	280.1 ± 67.3	23.9 ± 60.8	268.12 ± 59.8	35.88 ± 56	0.01*
HbA1c (%)	8.7 ± 1.3	8.3 ± 2.2	0.4 ± 1.92	8.1 ± 2.0	0.6 ± 1.76	0.1
HOMA-IR	3.6 ± 1.27	3.14 ± 0.23	0.46 ± 1.17	3.07 ± 0.34	0.53 ± 1.14	0.08
HOMA 2% B	68.16 ± 12.3	72.14 ± 14.2	3.98 ± 13.3	73.12 ± 16.8	4.96 ± 15	0.72
Fasting Insulin (µIU/ml)	40 ± 3.45	38.9 ± 7.5	1.1 ± 6.5	37.7 ± 8.9	2.3 ± 7.7	0.22
**Intervention Group**						
FPG (mg/dl)	253.2 ± 50.2	164.7 ± 37.8	88.5 ± 45.2	131 ± 29.9	122.2 ± 43.7	0.0001***
PPG (mg/dl)	312 ± 67.2	198.6 ± 59.3	113.4 ± 63.6	188 ± 31.3	124 ± 58.2	0.002*
HbA1c (%)	8.5 ± 2.0	7.4 ± 1.0	1.1 ± 1.73	6.9 ± 0.5	1.6 ± 1.8	0.01*
HOMA-IR	3.8 ± 1.06	3.74 ± 0.07	0.06 ± 1.03	2.75 ± 0.02	1.05 ± 1	0.002**
HOMA 2% B	66.7 ± 14.7	74.6 ± 16.8	7.9 ± 15.8	80.2 ± 17.8	13.5 ± 16.4	0.003**
Fasting Insulin (µIU/mL)	41.2 ± 3.9	39.6 ± 6.9	1.6 ± 5.99	30.5 ± 7.8	10.7 ± 6.7	0.001***

**Note:** The represented values were expressed as mean ± standard deviation changes. P-values were calculated based on repeated measures ANOVA for multiple comparisons.

**Table 3:** Changes in lipid parameters within the treatment groups.

**Standard Treatment Group**						
**Parameter**	**Baseline Visit**	**12 Weeks**	**Mean Difference**	**24 Weeks**	**Mean Difference**	***P*-value**
TC (mg/dl)	153.9 ± 18.8	152.4 ± 25	1.5 ± 22	150.5 ± 18.4	3.4 ± 19	0.06
LDL (mg/dl)	144.6 ± 11.8	147 ± 14.6	2.4 ± 13.2	135.5 ± 11.7	9.1 ± 11.7	0.01*
VLDL (mg/dl)	34.3 ± 3.55	32.9 ± 4.67	1.4 ± 4.22	33.6 ± 3.63	0.7 ± 3.5	0.08
HDL (mg/dl)	37.5 ± 3.25	36.1 ± 4.87	1.4 ± 4.17	39.2 ± 6.85	1.7 ± 5.4	0.03*
TG (mg/dl)	168.1 ± 13.8	162.2 ± 14.9	5.9 ± 14.37	158.3 ± 19.8	9.8 ± 17	0.01*
BMI (Kg/m^2^)	29 ± 1.4	31.1 ± 2.4	2.1 ± 2	31.9 ± 2.8	2.9 ± 2.64	0.77
**Intervention Group**						
TC (mg/dl)	156.2 ± 14.9	153.7 ± 18.8	2.5 ± 16.7	138.9 ± 17.6	17.3 ± 16.24	0.02*
LDL (mg/dl)	148.7 ± 14.11	131.5 ± 10.9	17.2 ± 13.01	128.6 ± 13.3	20.1 ± 13.7	0.002**
VLDL (mg/dl)	35.5 ± 6.12	32.3 ± 4.08	3.2 ± 5.37	30 ± 3.4	5.5 ± 4.7	0.01*
HDL (mg/dl)	39.5 ± 6.07	38.6 ± 5.42	0.9 ± 5.75	43.3 ± 4.8	3.8 ± 5.48	0.001**
TG (mg/dl)	164.9 ± 13.4	156.2 ± 14.3	8.7 ± 13.8	148.7 ± 16.4	16.2 ± 14.9	0.01*
BMI (Kg/m^2^)	31 ± 3.2	29.1 ± 1.2	1.9 ± 2.59	27.9 ± 1.9	3.1 ± 2.6	0.01*

**Note:** The represented values were expressed as mean ± standard deviation changes. *P*-values were calculated based on repeated measures ANOVA for multiple comparisons.

**Table 4:** Comparison of glycemic and lipid parameters between the treatment groups.

**Glycemic Parameters**								
**Parameter**	**Group**	**Baseline**	**12 Weeks**	**Between-group Difference**	***P* value** **(12 weeks)**	**24 Weeks**	**Between-group Difference**	***P* value** **(24 weeks)**
FPG (mg/dL)	Standard	230.8 ± 41.2	197.8 ± 25.7	33.1 ± 33	0.001***	164 ± 19.2	33 ± 26.2	0.001***
	Intervention	253.2 ± 50.2	164.7 ± 37.8			131 ± 29.9		
PPG (mg/dL)	Standard	304 ± 51.2	280.1 ± 67.3	81.5 ± 63.7	0.001***	268.1 ± 59.8	80.1 ± 5 1.8	0.001***
	Intervention	312 ± 67.2	198.6 ± 59.3			188 ± 31.3		
HbA1c (%)	Standard	8.7 ± 1.3	8.3 ± 2.2	0.9 ± 1.9	0.01*	8.1 ± 2.0	1.2 ± 1.5	0.009**
	Intervention	8.5 ± 2.0	7.4 ± 1.0			6.9 ± 0.5		
HOMA-IR	Standard	3.6 ± 1.27	3.14 ± 0.23	0.6 ± 0.2	0.09	3.07 ± 0.34	0.3 ± 0.4	0.08
	Intervention	3.8 ± 1.06	3.74 ± 0.07			2.75 ± 0.02		
Fasting Insulin (µIU/mL)	Standard	40 ± 3.45	38.9 ± 7.5	0.7 ± 7.2	0.3	37.7 ± 8.9	7.2 ± 11. 7	0.02*
	Intervention	41.2 ± 3.9	39.6 ± 6.9			30.5 ± 7.8		
HOMA2%B	Standard	68.16 ± 12.3	72.1 ± 14.2	2.5 ± 15.4	0.07	73.1 ± 16.8	7.1 ± 17. 2	0.01*
	Intervention	66.7 ± 14.7	74.6 ± 16.8			80.2 ± 17.8		
TC (mg/dL)	Standard	153.9 ± 18.8	152.4 ± 25.0	1.3 ± 22.6	0.1	150.5 ± 18.4	11.6 ± 18	0.001***
	Intervention	156.2 ± 14.9	153.7 ± 18.8			138.9 ± 17.6		
LDL (mg/dL)	Standard	144.6 ± 11.8	147 ± 14.6	15.5 ± 13.1	0.06	135.5 ± 11.7	6.9 ± 9.7	0.001***
	Intervention	148.7 ± 14.1	131.5 ± 10.9			128.6 ± 13.3		
VLDL (mg/dL)	Standard	34.3 ± 3.55	32.9 ± 4.67	0.6 ± 4.4	0.1	33.6 ± 3.63	3.6 ± 3.5	0.03*
	Intervention	35.5 ± 6.1	32.3 ± 4.1			30 ± 3.4		
HDL (mg/dL)	Standard	37.5 ± 3.25	36.1 ± 4.87	2.5 ± 5.2	0.02*	39.2 ± 6.85	4.1 ± 6.1	0.01*
	Intervention	39.5 ± 6.07	38.6 ± 5.42			43.3 ± 4.8		
TG (mg/dL)	Standard	168.1 ± 13.8	162.2 ± 14.9	6 ± 14.6	0.03*	158.3 ± 19.8	9.6 ± 18.4	0.01*
	Intervention	164.9 ± 13.4	156.2 ± 14.3			148.7 ± 16.4		
BMI (kg/m^2^)	Standard	29.0 ± 1.4	31.1 ± 2.4	2 ± 2.3	0.23	31.9 ± 2.8	4 ± 3.4	0.02*
	Intervention	31.0 ± 3.2	29.1 ± 1.2			27.9 ± 1.9		

**Note:** The represented values were expressed as mean ± standard deviation changes. *P*-values were calculated based on ANOVA.

## Data Availability

The necessary source data regarding this study is available upon reasonable request to the corresponding author.

## LIST OF ABBREVIATIONS

ACEi: Angiotensin Converting Enzyme Inhibitor
AMPK: AMP-Activated Protein Kinase
ARBs: Angiotensin Receptor Blockers
BMI: Body Mass Index
BP: Blood Pressure
CI: Confidence Interval
CTRI: Clinical Trials Registry of India
DAPA-HF: Dapagliflozin and Prevention of Adverse Outcomes in Heart Failure Trial
DAG: Diacylglycerol
EF: Ejection Fraction
FFA: Free Fatty Acids
FPG: Fasting Plasma Glucose
HbA1c: Glycated Hemoglobin
HDL: High-Density Lipoprotein
HF: Heart Failure
HFmrEF: Heart Failure with mildly reduced Ejection Fraction
HOMA-IR: Homeostatic Model Assessment of Insulin Resistance
HOMA2%B: Homeostatic Model Assessment of β-Cell Function
HPLC: High-Performance Liquid Chromatography
HSL: Hormone-Sensitive Lipase
IEC: Institutional Ethics Committee
IR: Insulin Resistance
lb-LDL: Large Buoyant Low-Density Lipoprotein
LDL: Low-Density Lipoprotein
LPL: Lipoprotein Lipase
PPAR-α: Peroxisome Proliferator-Activated Receptor Alpha
PPG: Postprandial Plasma Glucose
R^2^: Coefficient of Determination
sd-LDL: Small Dense Low-Density Lipoprotein
SGLT2: Sodium-Glucose Cotransporter 2
SGLT2i: Sodium-Glucose Cotransporter 2 Inhibitors
SPSS: Statistical Package for the Social Sciences
T2DM: Type 2 Diabetes Mellitus
TC: Total Cholesterol
TG: Triglycerides
UTI: Urinary Tract Infection
VLDL: Very Low-Density Lipoprotein

## STANDARDS OF REPORTING

CONSORT guidelines were followed.
